# Peripheral Ameloblastoma of Acanthomatous Variant: A Case Report

**DOI:** 10.31729/jnma.7739

**Published:** 2022-12-31

**Authors:** Ripu Singh, Sajeev Shrestha, Pujan Acharya, Ashish Shrestha

**Affiliations:** 1 Department of Dentistry, Rapti Academy of Health Sciences, Ghorahi, Dang, Nepal; 2 Department of Periodontology and Oral Implantology, College of Dental Surgery, B.P. Koirala Institute of Health Sciences, Dharan, Sunsari, Nepal; 3 Department of OraT Pathology, College of Dental Surgery, B.P. Koirala Institute of Health Sciences, Dharan, Sunsari, Nepal

**Keywords:** *ameloblastoma*, *case reports*, *histopathology*, *odontogenic tumour*

## Abstract

Ameloblastoma is an uncommon benign epithelial neoplasm of odontogenic origin that accounts for about 18% of the odontogenic tumour. It is a slow-growing, locally invasive but rarely metastatic tumour. Extraosseously occurring peripheral ameloblastoma is a rare variant that comprises about 2-10% of all ameloblastoma. We report a case of peripheral ameloblastoma in a 43-years old male patient affecting the mandibular canine-premolar region with a histopathological diagnosis of acanthomatous ameloblastoma. This case report emphasises the clinical, radiographic, and histological features of a rare variant that distinguishes it from the other similar appearing lesion on gingiva along with its various treatment modalities.

## INTRODUCTION

Ameloblastoma is a neoplasm of odontogenic epithelium, particularly of enamel organ-type tissue that has not undergone differentiation to the point of forming hard tissue.^1^ It is generally a slow-growing but locally invasive tumour that accounts for about 1% of all oral cysts/tumours of the jaw and 18% of odontogenic neoplasms.^2^ Peripheral ameloblastoma (PA) is also known as ameloblastoma of the gingiva or extraosseous ameloblastoma. Here we report a rare case of peripheral ameloblastoma of the acanthomatous variant in the mandibular canine-premolar region in a male patient treated with surgical excision.

## CASE REPORT

A 43-year-old male patient reported to the Department of Periodontology and Oral Implantology with a chief complaint of swelling in the left lower front region of the jaw for 3 years, progressive in nature. The past medical and family history was non-relevant. Intraoral examination revealed a localised, ovoidshaped swelling of size 1 x 1.5 cm extending from the attached gingiva to the depth of the buccal vestibule between the right lower canine and first premolar. On palpation, it was firm, fixed, non-fluctuant, and non tender. The contiguous teeth were vital to the cold test. Clinically, the involvement of the lingual aspect of the mandible was not appreciable. No fluid was aspirated from the lesion. Based on the patient's history, clinical examination, and chairside investigations, a peripheral-ossifying fibroma was considered a provisional diagnosis. Differential diagnoses of a benign odontogenic tumour, peripheral giant cell granuloma, and fibroma were made.

**Figure 1:**
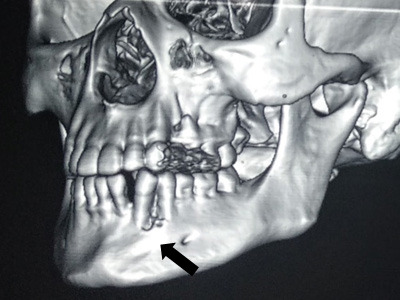
CT scan revealed a mild lytic lesion with an erosion of the alveolar cortex (indicated by a black arrow).

Radiographic evaluation with periapical and panoramic radiographs revealed missing 32 and slight displacement of the root of canine towards mesial aspect without root resorption. The computed tomography (CT) scan revealed a mild lytic lesion with an erosion of the alveolar cortex in the left lower canine-premolar region (Figure 1).

**Figure 2:**
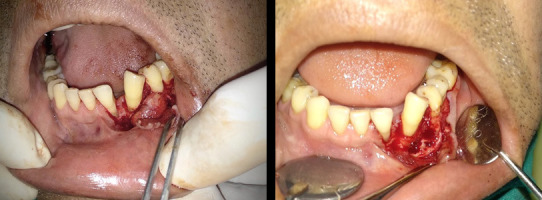
Intraoperative picture after the elevation of a mucoperiosteal flap and complete excision of the mass.

After consultation with the Department of Oral and Maxillofacial Surgery, an excisional biopsy of the mass was planned. Informed and written consent were taken and the patient was subjected to biopsy. Under local anaesthesia, an envelope mucoperiosteal flap was elevated and the underlying mass was then localised. The complete excision of the mass along with adequate alveolar margin was resected (Figure 2).

The operative site was then irrigated to remove any residual fragments and debris. The excised surgical specimen was sent for histopathological examination. The flap closure was done with sutures. Satisfactory postoperative healing was noted.

**Figure 3:**
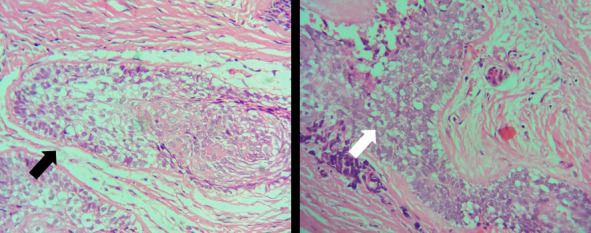
Histopathological picture showing follicle with squamous cell/ acanthomatous change (black arrow) and peripheral cells showing ameloblast celllike differentiation (white arrow) (Hematoxylin and eosin stain, 40x).

Histopathological examination of the specimen revealed odontogenic tumour islands of variable shapes and sizes lining with peripheral tall columnar ameloblast-like cells with palisaded hyperchromatic nuclei and central stellate reticulum-like cells. Multiple areas demonstrated squamous metaplasia of stellate-reticulum-like cells. Numerous tumour cords and islands exhibited hyalinization at the periphery along with a focal area of bony trabeculae with osteocytes. The connective tissue stroma was fibrous with collagen fibres arranged in bundles along with a few inflammatory cells and blood vessels of variable sizes (Figure 3).

The histopathological diagnosis of acanthomatous ameloblastoma was made. Clinical, radiographics, and histopathological findings finally led to the diagnosis of peripheral ameloblastoma of the acanthomatous variant in the mandibular canine-premolar region. A close, periodic clinical, and radiographic follow-up of the case was maintained. The most recent clinical revaluation at 18 months follow-up revealed no signs of recurrence at the treated site.

## DISCUSSION

According to the World Health Organization (WHO), ameloblastoma is defined as a benign but locally invasive neoplasm consisting of proliferating odontogenic epithelium located in a fibrous stroma. Despite its aggressive local behaviour, it rarely metastasizes to other organs.^3^ According to the WHO classification of odontogenic tumours in 2005, ameloblastomas were divided macroscopically into four types: solid/multicystic, extraosseous/peripheral, desmoplastic, and unicystic. However, in 2017 it was simplified to ameloblastoma, unicystic ameloblastoma, and extraosseous/peripheral types.^4^ Among these, peripheral ameloblastoma is an uncommon variant with a prevalence of 1-5%.^5^ The most probable sources of PA are; the remnants of dental lamina so-called "glands of Serres", odontogenic remnants of the vestibular lamina, the pluripotent cells of the basal layer of the mucosal epithelium, and the pleuripotent cells of the minor salivary gland.^6^

Similarly, various histological subtypes of ameloblastoma have been described, including follicular, plexiform, acanthomatous, granular, and basal cell types. Most literature showed that follicular ameloblastoma is the most prevalent histological variant (64.9%) followed by plexiform (13.0%), desmoplastic (5.2%), and acanthomatous (3.9%) varieties.^7^

PA has the peak incidence in the fifth to sixth decades of life while other intraosseous ameloblastomas usually present at a younger age.^8^ Slight male predilection has been reported for all forms of ameloblastomas including PA.^2^ While about 80% of ameloblastoma occur in the mandible with 70% being located in the molar-ramus area, the lingual aspect of the mandibular premolar region is reported as the most commonly involved site of PA that accounts for 32.6% of all sites.^9^ These data were consistent with our case except for the involvement of the buccal aspect of the mandible rather than the lingual. Some other unusual sites for PA include buccal mucosa, the base of the tongue, and the floor of the mouth.^8^

Similar to all other cysts and bone tumours, the diagnosis of ameloblastoma requires appropriate clinical and radiological examination. Clinically PA presents as a slow-growing exophytic mass arising from the soft tissue of the tooth-bearing region of the jaw and therefore may be misinterpreted as other peripherally occurring lesions like pyogenic granuloma, peripheral ossifying fibroma, and peripheral giant cell granuloma. PA usually is an indolent tumour that doesn't invade the underlying bone and therefore does not present any distinct radiographic evidence as in our case. CT and magnetic resonance imaging (MRI) usually depict PA as a well-demarcated mass but do not demonstrate invasion of the jaws and adjacent muscles. However, a mild erosion or local depression on the cortical bone without reaching the bone marrow known as cupping or saucerization has been reported to occur occasionally which was detected even in our case. An interesting issue regarding the histology of PA is that some authors consider PA and basal cell carcinoma (BCC) as the same lesion as both of them share some peculiar histopathologic characteristics such as basal cells and their island arrangements.^10^ However, immunohistochemical analysis differentiates these two entities as patterns of positivity for cytokeratins are different between PA and BCC.

Various histopathological patterns are exhibited by PA with the tendency for acanthomatous variant as in our case.^8^ According to the WHO classification, the term acanthomatous ameloblastoma should be applied when extensive squamous metaplasia, sometimes with keratin formation, is found within the islands of tumour cells as evident in our case.

Treatment modalities for ameloblastoma include both radical and conservative surgical excision, radiation therapy, or a combination of surgery and radiation. The basic principle for the cure remains complete removal of the neoplasm, regardless of how it is accomplished. Resection with a safety margin is reported as the best and the most preferred method in the treatment of ameloblastomas and the same was done in our case. A study reported a recurrence of 9% following the treatment of PA. Although rare, malignant transformation and metastasis of PA have also been reported.^8^

Peripheral ameloblastoma although benign neoplasm with no aggressive behavior or invasive potential unlike intraosseous ameloblastoma, owing to the possibility of its recurrence and malignant transformation, a long-term follow-up is recommended even after the definitive treatment.

In the present case, although three-dimensional computed tomographic imaging, as well as complete surgical excision with adequate safety margin, further confirmed with histopathological examination and 18 months follow-up was done, longer clinical and radiographic follow-up was deemed necessary as there was even bony involvement in the form of cortical erosion.

Early diagnosis of an uncommon but locally aggressive odontogenic tumor of the jaw like ameloblastoma is paramount for definitive treatment and minimizing reconstructive challenges. However, mere clinical and radiographic presentation of early-stage ameloblastoma may not be pathognomic as in our case of peripheral ameloblastoma. Therefore, a histopathological interpretation is highly indicated not only for the diagnosis of such mass but also for the identification of its histological variant based upon which its prognosis, definitive management, and treatment outcome can be determined.

## References

[1] Nakamura N, Mitsuyasu T, Higuchi Y, Sandra F, Ohishi M (2001). Growth characteristics of ameloblastoma involving the inferior alveolar nerve: a clinical and histopathologic study.. Oral Surg Oral Med Oral Pathol Oral Radiol Endod..

[2] Reichart PA, Philipsen HP, Sonner S (1995). Ameloblastoma: biological profile of 3677 cases.. Eur J Cancer B Oral Oncol..

[3] Madiedo G, Choi H, Kleinman JG (1981). Ameloblastoma of the maxilla with distant metastases and hypercalcemia.. Am J Clin Pathol..

[4] Lee SK, Kim YS (2013). Current concepts and occurrence of epithelial odontogenic tumours: I. Ameloblastoma and adenomatoid odontogenic tumour.. Korean J Pathol..

[5] Vezhavendhan N, Vidyalakshmi S, Muthukumaran R, Santhadevy A, Sivaramakrishnan M, Gayathri C (2019). Peripheral ameloblastoma of the gingiva.. Autops Case Rep.

[6] Baden E, Doyle JL, Petriella V (1993). Malignant transformation of peripheral ameloblastoma.. Oral Surg Oral Med Oral Pathol..

[7] Adebiyi KE, Ugboko VI, Omoniyi-Esan GO, Ndukwe KC, Oginni FO (2006). Clinicopathological analysis of histological variants of ameloblastoma in a suburban Nigerian population.. Head Face Med.

[8] Beena VT, Choudhary K, Heera R, Rajeev R, Sivakumar R, Vidhyadharan K (2012). Peripheral Ameloblastoma: a case report and review of literature.. Case Rep Dent..

[9] Philipsen HP, Reichart PA, Nikai H, Takata T, Kudo Y (2001). Peripheral ameloblastoma: biological profile based on 160 cases from the literature.. Oral Oncol..

[10] Simpson HE (1974). Basal-cell carcinoma and peripheral ameloblastoma.. Oral Surg Oral Med Oral Pathol..

